# Positive and Negative Impacts of Caring among Adolescents Caring for Grandparents. Results from an Online Survey in Six European Countries and Implications for Future Research, Policy and Practice

**DOI:** 10.3390/ijerph17186593

**Published:** 2020-09-10

**Authors:** Sara Santini, Marco Socci, Barbara D’Amen, Mirko Di Rosa, Giulia Casu, Valentina Hlebec, Feylyn Lewis, Agnes Leu, Renske Hoefman, Rosita Brolin, Lennart Magnusson, Elizabeth Hanson

**Affiliations:** 1 Centre for Socio-Economic Research on Aging, IRCCS INRCA-National Institute of Health and Science on Aging, Via Santa Margherita 5, 60124 Ancona, Italy; s.santini2@inrca.it (S.S.); b.damen@inrca.it (B.D.); 2 Unit of Geriatric Pharmacoepidemiology and Biostatistics, IRCCS INRCA-National Institute of Health and Science on Aging, Via Santa Margherita 5, 60124 Ancona, Italy; m.dirosa@inrca.it; 3 Department of Psychology, University of Bologna, viale Berti Pichat 5, 40127 Bologna, Italy; giulia.casu3@unibo.it; 4 Faculty of Social Sciences, University of Ljubljana, Kardeljeva pl. 5, 1000 Ljubljana, Slovenia; Valentina.Hlebec@fdv.uni-lj.si; 5 Faculty of Health Sciences, University of Ljubljana, Zdravstvena pot. 5, 1000 Ljubljana, Slovenia; 6 School of Education and Social Work, University of Sussex, Essex House 121, Brighton BN1 9QQ, UK; F.M.Lewis@sussex.ac.uk; 7 Careum School of Health, University of Applied Sciences, Pestalozzistrasse 3, 8032 Zürich, Switzerland; Agnes.Leu@careum.ch; 8 Medical Faculty, Institute of Biomedical Ethics, University of Basel, Klingelbergstrasse 61, 4056 Basel, Switzerland; 9 The Netherlands Institute for Social Research (SCP), Postbus 16164, 2500 BD De Hague, The Netherlands; r.hoefman@scp.nl; 10 Department Health and Caring Sciences, Linnaeus University, SE-39182 Kalmar, Sweden; rosita.brolin@lnu.se (R.B.); lennart.magnusson@lnu.se (L.M.); elizabeth.hanson@lnu.se (E.H.); 11 The Swedish Family Care Competence Centre, Strömgatan 13, SE-39232 Kalmar, Sweden

**Keywords:** adolescent young caregivers, grandparents, intergenerational relationships, older people, online survey, negative caregiving outcomes, positive caregiving outcomes

## Abstract

Although up to 8% of European youngsters carry out high-intensity care for a family member, adolescent young carers (AYCs), especially those caring for their grandparents (GrPs), remain an under-researched group. This study aimed at addressing the current knowledge gap by carrying out an online survey in Italy, the Netherlands, Slovenia, Sweden, Switzerland, and the United Kingdom. The analysis included a final sample of 817 AYCs aged 15–17 years old. AYCs of grandparents (GrPs) were compared to AYCs of other care recipients (OCRs), in order to identify any difference in positive and negative caregiving outcomes and exposure factors between the two groups. Linear or logistic regression models were built, and multivariate analyses were repeated, including a fixed effect on the country variable. AYCs of GrPs experienced more positive caregiving outcomes than AYCs of OCRs across all six countries. Being female or non-binary, and having a migration background, were associated with more negative outcomes, regardless of the relationship with the care recipient. Further research on intergenerational caregiving outcomes is recommended for shaping measures and policies, which preserve the intergenerational emotional bonds, whilst protecting AYCs from inappropriate responsibilities, undermining their mental health and well-being.

## 1. Introduction

In Europe and beyond older people with long-term care (LTC) needs are cared for mainly by family carers. The latter are a silent workforce, representing the backbone of the European LTC systems, especially in countries where there is a paucity of formal care services for older/disabled people [1,2]. The typical family carer is a middle-aged adult, usually a woman, who often takes care of an older relative with one or more chronic diseases, cognitive problems, or physical and/or mental disability [3,4]. Nonetheless, children and adolescents under 18 may also find themselves in the situation of carrying out significant or substantial caring responsibilities for an older, sick, or disabled family member, frequently on a regular basis, and may include a grandparent, and/or other care recipients within the household. Such young people are termed “young carers” or “young caregivers” [5,6] and they rarely receive training for their caring role [7]. Within the group of young carers (YCs), youngsters in the middle adolescence phase, i.e., aged 15–17 and in the critical transition period from adolescence to adulthood [8], are included, to whom we refer in the context of this paper as adolescent young carers (AYCs).

This study aims at shedding light on AYCs of grandparents (GrPs), who represent an under-researched group of YCs. In light of the paucity of literature focusing on this particular group of AYCs, we referred to research on YCs. Even in this case however, studies remain relatively scarce, especially in continental Europe, partly because of the low level of self-recognition of YCs [9,10]. Moreover, the lack of consistency, regarding the definitions of YCs across the different studies, makes it difficult to carry out research with, and formally identify, YCs [7,11,12]. Nevertheless, it is calculated that between 2% and 8% of children in developed countries have caring responsibilities within their families [13,14], carrying out a wide variety of tasks [15,16]. For example, YCs help GrPs with personal hygiene and meal preparation [17], and in performing instrumental activities of daily living, companionship, and emotional support [18]. Several studies, mainly focused on multigenerational families [19], classified YCs as primary [20,21] and auxiliary carers [22], depending on whether they were in the frontline or covered a subsidiary role in providing care. The available literature on YCs of GrPs highlights that they may assist their older relatives because other adults are unavailable or unwilling to provide care [23,24,25,26,27], as a way of contributing to the family [28], and in response to the request of parents [17], especially when the latter provide a high number of hours of assistance and combine care with paid work [24]. Other studies focusing on YCs of GrPs highlight that the stronger and closer the relationship between GrPs and grandchildren, the more likely it is that young people take on the role of carers, to reciprocate the love and nurturance received when they were young children [23,24,29].

Caring for a family member (not only an older family member) is associated with both positive and negative consequences on youngsters’ lives [30]. Positive caregiving outcomes include greater resilience [31], especially when the care demand is not too high [32], increased maturity [33], and empathy [34]. With regard to AYCs [18], and YCs of GrPs [35], they may develop new relational skills, thereby enhancing their self-image and self-efficacy, and develop a positive and sensitive representation of ageing [36], especially when they can rely on sufficient practical and psychological support from other family members and friends, and/or on formal social and healthcare services [37].

Nevertheless, taking care of a family member can also have negative caregiving outcomes, especially for adolescents who have still to define a personal sense of identity, adopt a subjective value system, and re-negotiate relationships with parents as carers, during this developmental and transitional phase to adulthood [8]. A young caregiving role may indeed exacerbate the risk of experiencing mental health problems [38], health inequalities during the life course [5,12,39,40], poor well-being [41,42], restrictions in education and employment opportunities [43,44], as well as stigma leading to social isolation [45], frustration [35], and stress [25]. Furthermore, the recent Covid-19 pandemic, and the lockdown imposed by European governments as a response to the outbreak, had a dramatic impact on the mental health and well-being of family carers of all ages [46], and AYCs especially experienced a worsening of their mental health and social isolation [47].

This paper endeavors to contribute to the knowledge on AYCs of GrPs by reporting the results of a survey carried out within the project “Psychosocial support for promoting mental health and well-being among adolescent young caregivers in Europe” (ME-WE), funded by the European Union (H2020; 2018-2021). One of the overall project objectives was to increase and systematize the knowledge concerning AYCs’ situation across Europe. The project activities included a cross-national survey on AYCs, carried out in 2018, in six European countries (Italy, the Netherlands, Slovenia, Sweden, Switzerland, and the United Kingdom). The ME-WE survey involved a large sample of 15–17 AYCs, thus providing a unique cross-European comparison of AYCs of GrPs, and allowing the comparability of results of large samples, as recommended by researchers in the field [7,48]. Starting from this baseline, this study specifically aimed at answering the following research questions: (1) Is there any difference in positive and negative caregiving outcomes between AYCs of GrPs and AYCs of other care recipients (OCRs)? (2) Which are the exposure factors to positive and negative caregiving outcomes among AYCs of GrPs, in comparison to AYCs of OCRs?

## 2. Materials and Methods

### 2.1. Participants’ Inclusion Criteria and Recruitment Strategies

The study was carried out via an online survey between March and October 2018, and it targeted adolescents aged 15–17. The data collection sheet was elaborated in English and translated into the study countries’ languages. Since this was the first international and large sample survey on AYCs in Europe aiming at mapping this phenomenon, the only criteria for being included in the survey was being aged between 15–17 years and being available to fill in the questionnaire. Within the samples of the six countries, AYCs were identified using two anchoring questions (see Measures section) and afterwards, a sub-sample of AYCs of GrPs was compared to AYCs of OCRs, e.g., parents, siblings, friends. Although caring for a family member (often cohabiting) can entail a heavier care burden than caring for a close friend, the authors decided to consider ‘not-grandparent family members and close friends’ within the same group (and not to make multiple sub-groups of care recipients, e.g., parents, siblings, etc.) with the aim of focalizing the influence of the specific intergenerational relationship between AYCs and GrPs, on caring outcomes. Hence, the researchers were interested in the type of kinship between the AYC and the cared for person, in particular the older care recipient, and in understanding the influence that this kind of relationship might have on caring outcomes, rather than in the number of people AYCs were caring for. Whilst, the authors acknowledge that the latter could be important information for quantifying the caregiving burden, it was not the main topic of this article. In order to address the disparity of awareness and knowledge around the issue of AYCs, at the level of the participating countries, the recruitment strategies were tailored to target AYCs aged 15–17 in the most efficient way. In Italy, Slovenia, and Switzerland, respondents were mainly recruited among high school students. In the Netherlands, participants were recruited through schools, care support centers, patient and carer organizations, and social media channels. In Sweden, the youngsters were reached in schools as well as in social care and civil society organizations. In the United Kingdom (UK), where there is a high level of awareness on the topic and a wide availability of specialized non-governmental organizations for young carers (called “young carers projects”), the recruitment took place mainly through young carers projects and a small number of schools. Where data was collected in schools, all pupils in a class were invited to fill in the survey.

### 2.2. Data Collection Procedure

The survey was carried out using the 1ka online platform, in order to guarantee participants’ anonymity and privacy, on different types of electronic devices, e.g., personal computer, laptop, tablet, and smartphone. In some cases, data collection in school settings required a paper and pencil data collection mode, due to the large number of participants encountered in school classrooms and the limited availability of electronic devices. The paper and pencil data were transferred to online platform by experienced researchers belonging to national project research teams. The original English version of the questionnaire was translated into the national languages independently by two researchers per national team, and revised by a third national team member. To ensure that the national cultures did not unduly influence the interpretation of the core concepts, the latter were introduced with a brief explanation in the questionnaire, in simple and non-technical language to be understandable by the youngest respondents and for avoiding stigmatization. For example, the concept of “caring” was explained as follows: Sometimes young people like yourself provide support and assistance to their family members or close friends with health-related conditions, we call this support and assistance “caring”. In some countries, researchers were available to address any questions raised by participants, and teaching staff could approach researchers by e-mail to ask survey-related questions.

### 2.3. Ethics

Ethics approval was obtained from relevant ethical review boards in all six partner countries prior to the start of the study (Spring 2018). All respondents were recruited on a voluntary basis in accordance with the Declaration of Helsinki (1964; 2013) [49]. By filling in the questionnaire, respondents gave their informed consent to take part in the study. An information letter and the first page of the online questionnaire made it clear that AYCs’ participation was voluntary and that they could withdraw at any time without any explanation. Informed consent was also secured from parents/legal guardians, in accordance with applicable national legislation and institutional guidance. The data were processed in full compliance with both national laws on data protection and the General Data Protection and Regulation (EU 2016/679; Regulation, G.D.P.R., 2016) [50] to guarantee the respondents’ anonymity and privacy. No data was collected that could lead to any specific individual being identified, in order to fully protect the youngsters’ anonymity.

### 2.4. Measures

The questionnaire comprised four sections: demographic and caregiving information; impact of caring on education and available support services; open-ended questions for collecting suggestions on needs and useful supports for AYCs and feedback on questionnaire compilation. The first section began with questions about age, gender, country of birth, nationality, living condition, migration background, family composition, and caregiving hours per day. In order to evaluate the effects of caregiving activity on younger people, the following self-reported measures were applied: “Multidimensional Assessment of Caring Activities” (MACA-YC18) [30], “Positive and Negative Outcomes of Caring” (PANOC-YC20) [30], “KIDSCREEN-10” [51,52], and “Health problems in connection with caring”, which was an ad hoc developed single-item measure.

In order to identify AYCs among all the surveyed adolescents, a question about a family member or friend having a health related-condition, such as physical disability, mental illness, addiction, or other health-related condition, was asked, followed by an additional anchoring question, i.e., “Do you provide care, support, or assistance to a family member or friend because of their health-related condition?”. It was followed by questions for defining caregiving hours per day and profiling the relationship to the care recipient/s (e.g., mother, father, grandmother, grandfather, boyfriend, classmate), their health-related condition and level of impairment. All respondents then replied to both the MACA-YC18 and PANOC-YC20, and to the item on health problems in connection with caring.

MACA-YC18 is an 18-item self-report questionnaire asking young people about the type and frequency of their caring activities (items are rated on a 3-point scale where “never” = 0, “some of the time” = 1, and “a lot of time” = 2). An overall score is calculated ranging from 0 to 36. PANOC-YC20 is a 20 item self-report measure that provides indexes of positive and negative outcomes of care provision, each ranging from 0 to 20. Each item is rated on a 3-point scale: “never” = 0, “some of the time” = 1, and “a lot of the time” = 2. KIDSCREEN-10, a 10-item measure of the health-related quality of life standard, ranging from 10 to 50 was also applied. Each item is answered on a 5-point intensity or frequency response scale. AYCs were also asked to report if they suffered from physical (e.g., back pain, headache, muscle tension), psychological (e.g., anxiety, depression), or other health problems in connection to care activity provided by answering a multiple-choice question.

The second section of the questionnaire included dichotomous and multiple-choice questions about the impact of caregiving on education, and the support available for AYCs and their families, both formal (e.g., statutory agency/governmental supportive programs, services, and state monetary benefits) and informal (e.g., help from other family members, friends, neighbors). The third and fourth sections of the questionnaire respectively included open ended questions aimed at collecting AYCs’ emotions, difficulties, and suggestions for improving available support. The qualitative data is currently being analyzed both at national and cross-country level and, due to the rich amount of information they provide, they will be the subject of dedicated future publications arising from the ME-WE project.

### 2.5. Data Analysis

Preliminary analyses included test of differences between the two groups (i.e., AYCs of GrPs, and AYCs of OCRs) using t-test for continuous variables (i.e., scores on the MACA-YC18, PANOC-YC20 Negative, PANOC-YC20 Positive, KIDSCREEN-10) and chi-square tests for the dichotomous variable (i.e., self-reported health-problems in connection to the caring role). Among all outcomes, only those statistically significant in the previous analysis were kept for the multivariate analysis. Linear or logistic regression models (depending on the type of dependent variable) were built to estimate the association between caring for GrPs and health and well-being outcomes in AYCs, taking into account potential confounders (age, gender, hours a day spent providing care, country of birth, formal services). Finally, multivariate analyses were repeated including a fixed effect regression model on country variable. A 2-tailed *p* value <0.05 was considered significant. In order to avoid heteroskedasticity issues, and to accept the constant variance assumption, robust standard errors were applied using the White procedure. Moreover, in order to detect collinearity of the regressors with the constant, variance inflation factors (VIFs) were calculated, confirming that no collinearity issue could be raised. Data were analyzed using STATA version 15.1 (StataCorp, College Station, TX, USA).

## 3. Results

**Figure 1:**
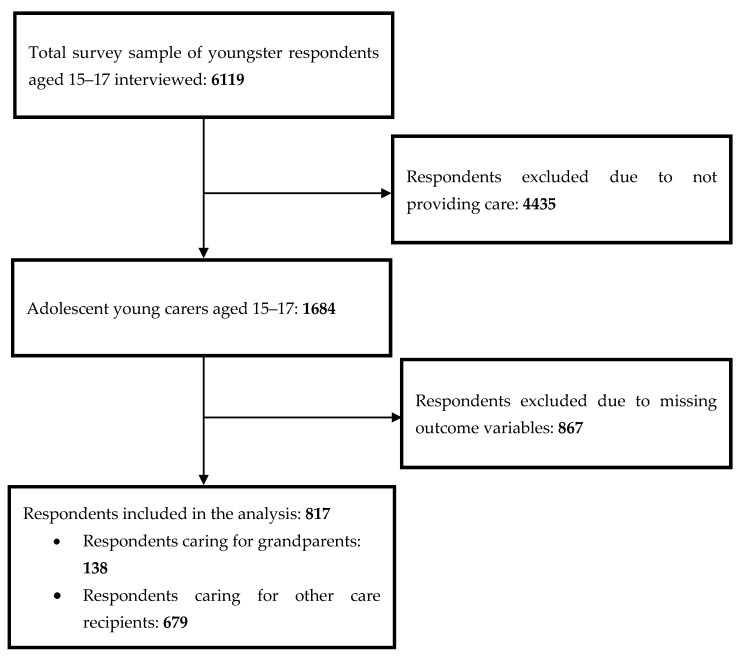
Procedure for the selection of the adolescent young carers (AYCs).

Out of 6119 youngsters aged 15–17 surveyed, 1684 (27.5%) were identified as AYCs by answering to the filter questions. Participants who did not respond to one or more outcome measure(s) were excluded from the analyses. Thus, the final sample comprised 817 AYCs (Figure 1).

Out of 817 AYCs, 138 (16.9%) were caring for a GrP (Table 1). The AYCs surveyed were mainly female (72.4% vs. 24.7% male, while 2.9% identified themselves as transgender/non-binary) and a similar ratio can be seen among both AYCs of GrPs (76.1% female, 21% male, and 2.9% transgender/non binary) and AYCs of OCRs (71.5% female, 25.5% male, and 3% transgender/non-binary). Within the whole sample, 7.2% of AYCs aged 15 and 46.4% of those aged 16–17 respectively were providing care to a GrP, while 13.8% of respondents aged 15, 43.8% of those aged 16, and 42.4% of those aged 17 cared for OCRs. The highest share of AYCs of GrPs, i.e., 36.3%, was from Italy, while 28.3% was from Slovenia, and the remaining 35.4% was distributed across the other surveyed countries (where the percentage of AYCs of GrPs was ≤10.1%).

A total of 92.5% of the whole sample (i.e., AYCs of GrPs and of OCRs), 93.5% of the AYCs of GrPs, and 92.3% of the AYCs of OCRs were born in their country of residence. While, 39% of AYCs of OCRs could count on formal and public care services to support them/their families (e.g., state care allowance and home care services), in comparison to 26.5% of AYCs of GrPs (*p* = 0.005). AYCs of OCRs reported caring four hours a day on average, about one hour more than AYCs of GrPs, (2.9 h a day on average).

### 3.1. Is There Any Difference in Positive and Negative Outcomes of Caregiving between AYCs of GrPs and AYCs of OCRs?

The descriptive statistics of the outcome variables are reported in Table 2. AYCs of OCRs and AYCs of GrPs did not differ in the amount of caring activities (MACA-YC18) or in health-related quality of life (KIDSCREEN). In the PANOC-YC20, AYCs of OCRs reported significantly higher negative outcomes (e.g., feeling stressed, lonely, upset, sad, exhausted) and significantly lower positive outcomes (e.g., feeling that I’m helping, closer to family, feeling that I am learning useful things, feelings of self-efficacy) than AYCs of GrPs.

### 3.2. Which Are the Exposure Factors to Positive and Negative Caregiving Outcomes and Health Problems among AYCs of GrPs, in Comparison to AYCs of OCRs?

Table 3 shows the results from the linear regression and the logistic regression between the exposition variable (in column) and the two outcome variables, PANOC-YC20 and Health problems, in connection to caring responsibilities (in the rows). In this table and in the following, MACA-YC18 and KIDSCREEN-10 are not included, due to the lack of statistical significance at multivariate level or already at descriptive level.

Caring for GrPs was associated with lower negative and higher positive outcomes, while it was unrelated to the outcome health problems due to caregiving. The age of AYCs is not associated with caregiving outcomes or health status. Being female, or transgender/non-binary, AYCs is positively associated with experiencing both more negative outcomes of caring and health problems due to caregiving, compared to male AYCs. The positive outcomes of care variable is not associated to gender. The greater the number of hours of assistance per day, the more likely that AYCs report negative outcomes. The amount of hours devoted to care is associated with the occurrence of health problems: a one-unit increase in hours of assistance per day was associated with a 6% increase in the odds of reporting health problems due to caregiving. Moreover, having a migrant background was associated with greater negative outcomes of care, but was not associated with the positive ones, nor with health problems.

AYCs (of GrPs) living in families who could count on formal care services reported greater negative outcomes, and were more likely to report health problems compared to AYCs, who could not count on formal care services. The group of AYCs who did not know if their family could count on formal services reported more negative outcomes.

Table 4 shows what happens when the country variable is included in the regression model. First, there is no longer any statistical significance between AYCs of OCRs and AYCs of GrPs, for both negative and positive outcomes, and for health problems related to care. Second, age continues not to be associated with positive or negative caregiving outcomes or health problems related to care. On the contrary, gender remains a variable influencing caregiving outcomes. Indeed, female and transgender/non-binary AYCs are exposed to more negative outcomes and more health problems than males.

The amount of daily care hours continues to be associated with both negative outcomes and health problems and having a migrant background remains associated with negative outcomes. As also reported in Table 3, the usage of formal services is associated with more negative outcomes and health problems in connection to providing care. The effect within the “don’t know” group instead disappears when including country in the model. From a cross-country perspective, by taking Switzerland as a benchmark, Swiss AYCs of GrPs are almost three times as likely (1/0.35) as those from the Netherlands to report health problems related to care. In Italy and Slovenia caring for a GrP is associated with less negative (*p* = 0.001 in both countries) and more positive outcomes (*p* = 0.069 and *p* = 0.022 respectively) compared to Switzerland. No statistically significant differences were found between AYCs living in Sweden or in the UK, compared to those living in Switzerland.

## 4. Discussion

The purpose of this study was to identify any difference/s in positive and negative outcomes of caregiving, and the exposure factors to these outcomes, among AYCs of GrPs, in comparison to AYCs of OCRs, in order to better understand the specific situation of adolescents who care for their GrPs on a regular basis. Among the AYCs of GrPs in our sample, the majority lived in Italy and in Slovenia rather than in the other study countries. This finding could be due to multiple factors, such as the lack of formal LTC services available for older people, the family oriented welfare system, and the high percentage of older people among the overall population in these countries [1,2]. It can be argued that in Italy and Slovenia, there is not a fully developed LTC system which can ensure formal home care services that wholly supports older people in need of daily and constant assistance [53,54]. Thus, adolescents may be asked by parents, in the role of family carers, to help with caregiving for their GrPs to counteract the dearth of public formal care services.

The lack of statistically significant differences between AYCs of GrPs and of OCRs, in the MACA-YC18 and in the KIDSCREEN-10, suggests that the type of kinship relationship with the care recipients (i.e., GrPs or OCRs) does not affect either the caring activities (typology and regularity) carried out by AYCs nor their health related quality of life. Conversely, caring for a GrP can decrease the negative outcomes, e.g., frustration and sense of inadequacy [25,35], mental health problems [38], and poor well-being [41,42]. Furthermore, it can influence the development of positive outcomes related to care (e.g., new relational skills, resilience, maturity, and empathy), thus confirming previous literature relating to YCs in general [18,31,33,34].

The mitigation of the negative caregiving outcomes, in the sub-group of AYCs of GrPs, could depend on the quality and strength of the relationships between GrPs and AYCs, or alternatively, on the high level of intergenerational cohesion they have experienced in their household [23,24,29,36].

Moreover, the onset of negative feelings was higher among AYCs carrying out greater amounts of caregiving, thereby suggesting that, despite caring for a GrP, bringing with it positive emotions, caring for an excessive number of hours, can nevertheless be potentially detrimental to AYCs’ overall health and well-being. It is also worth mentioning that, negative outcomes and the risk for health problems related to care continued to be higher among female and transgender/non-binary when the country variable was added, even if the statistical significance was mitigated. This might mean that from a cross-country perspective, females are obliged to provide care more than males, regardless of the typology of welfare regime, thus exposing girls to a higher risk of experiencing negative feelings and health problems related to caregiving provision [4]. Moreover, the survey data resulting from transgender/non-binary AYC respondents are in line with other studies in which transgender/non-binary young people were found to be more at risk of emotional distress in association with their caring role than cisgender and heterosexual AYCs [55].

AYC respondents with a migrant background were observed to be more likely than other AYCs to experience negative feelings in connection with caregiving, supporting previous literature on YCs [56]. This is seen to be likely due to both societal and cultural barriers to formal services [57], and a limited access to them [58].

This study found that receiving formal support is associated with more negative outcomes and a higher risk of health problems for AYCs of GrPs. This may depend on the fact that GrPs can access formal services because the state formally recognizes older people’s need for support (even if to varying extents per country), and it is precisely this need which can entail a greater care burden for AYCs (which is often associated with the severity of the care recipient’s health condition), thus influencing the onset of negative family caregiving outcomes. Conversely, the needs of AYCs are not explicitly recognized in social and family policies in many countries, and in this way they remain largely ignored by policy makers and service providers alike, at national and local levels [7,11,12,13,59].

Notably, when we considered the country variable, the statistical significance between AYCs of OCRs, and AYCs of GrPs, for the outcome variables disappeared, and the association of the outcome variables with migrant background and use of formal services was mitigated. To proof the reasons of this mitigation, data were analyzed by clustering countries according to the classification of countries by level of awareness, and development of policies and services for AYCs, elaborated by Leu and Becker [13]. Nevertheless, this analysis did not show any statistical significance across countries. This result suggests that the perception of positive and negative outcomes of caregiving could also be driven by cultural patterns [60], representations of and meanings given to illness and long-term health conditions [61], intergenerational family ties cohesion [23,24,29,36], perceived social support [62], self-recognition of the role of carer, and trust in social and health services [58]. The dearth of questions monitoring the above variables can be considered a limitation of the study. These variables indeed might have provided more details for understanding the influence of cultural patterns and family values on AYCs perception of caregiving.

Other limitations are the convenience sampling strategy, which did not allow for the generalization of results to all European AYCs, and the lack of a question asking the exact age of the care recipient. Moreover, although data on caring burden were partly collected by a question on the number of caring hours per day, the questionnaire did not include a question asking for the number of respondents’ care recipients. Furthermore, the high number of missing values in the outcome variables considerably reduced the number of AYCs to include in the analysis. This could have several explanations. First, some youngsters could have found the questionnaire excessively lengthy, and so they might have decided to skip some questions. Moreover, others could have felt uncomfortable in sharing their caregiving experience and/or did not self-recognize themselves in the questionnaire options, as this is a possibility with adolescent respondents of online surveys [63].

Despite these limitations, this study achieved an important goal, i.e., the comparability of findings on a large sample of AYCs, and from a European, cross-country perspective. This is indeed, the first large-scale international study on AYCs aged 15–17 years, and, to the best of our knowledge, the first cross-national study comparing AYCs of GrPs with AYCs providing care to OCRs. Hence, the findings can contribute to enriching the debate on this topic, orienting future policies and research.

Indeed, the outcomes of this study may inspire a number of areas for future research. First, since the perception of negative caregiving outcomes could be influenced by the strength of the intergenerational relationship between grandchild and GrP, such a relationship and the cohesion of the intergenerational ties within the household environment merit attention in future research, foreseeing specific questions on this aspect. Moreover, more research on AYCs from a gender identity perspective would help identify the frailties of female and transgender/non-binary AYCs, and mitigate negative repercussions on AYCs’ mental health and well-being [38,41,42,55].

Further research on black, asian, and minority ethnic (BAME) AYCs, and on family caregiving in second generation migrant families, is needed in order to provide an evidence-based interpretation of the negative caregiving outcomes experienced by AYCs with migration background. Future studies are also welcome, especially in countries where migrants are largely employed in the formal sector, and (the vast majority) by families themselves [64,65,66,67], to determine whether within migrant families, adolescents are asked to provide care to frail family members because their parents, mostly mothers, are employed as migrant care workers, thus becoming a sort of in-country “left behind” children [68].

In light of the possible influence of cultural factors on the caregiving outcomes highlighted by this study, country-specific research, as well as further cross-national studies on AYCs, are recommended. The first, exploring the cultural factors influencing the perception of caregiving outcomes, which may contribute to raising the awareness around the presence of AYCs, at national and local levels, and inspire policies and responsive support measures [7,13]. The second, i.e., cross-national studies, may allow: (a) providing further evidence on AYCs even in the mid-term, by comparing countries belonging to different welfare regimes; (b) the exchange of good practices; (c) the harmonization of European policies, in order to guarantee the same rights and protection afforded to all the European AYCs, in line with the recent recommendations of Joseph et al. [7]. This process should be developed within a framework aimed at supporting all informal carers across Europe, where AYCs are one of the several categories of people providing care that need special attention, dedicated laws, and regulations for sustaining their efforts, with a homogeneous set of rights and services available, in keeping with the UN Convention on the Rights of the Child [69]. This appears to be increasingly relevant in light of the impact of the recent (and ongoing) Covid-19 pandemic on family carers of all ages, including AYCs, who suddenly felt alone in their homes, dealing with family members in need of care due to old age, chronic illness and/or disability, and with a temporary reduction of home care based services [46,47].

Finally, further research, specifically focusing on AYCs of older people, is required, employing mixed methodologies. This is in order to capture the specific needs and preferences of such AYCs, and to gain useful input for designing, and putting into place, evidence-based practices and services that can effectively support this particularly hidden category of carers.

Of potential relevance here is also research and development work on intergenerational relationships, which could in turn help to shape specific policies aimed at combining the needs and interests of both older and younger population cohorts, in compliance with the European Union policy line on active ageing and intergenerational solidarity [70]. This intergenerational solidarity-oriented policy could be translated into practice by means of intergenerational programs, such that the generations can have more opportunities for meeting, collaborating, and exchanging experiences [71]. In a preventive perspective, these kinds of interventions could help to stimulate positive representations of ageing among youngsters and adolescents. In doing so, helping them to draw on positive insights on ageing, should they later decide to help care for their own grandparent/s.

This study has shed light on a group of carers, AYCs of GrPs, that are largely hidden to both policy makers and professionals alike. We argue that by adopting a family-focused approach in health and social care policies and practices for older people, this would enable front-line practitioners to actively reach out to, and subsequently identify, at an early stage, all family members, including AYCs, involved in the regular help, support, and/or care of a frail older relative. This being the crucial first-step towards recognition of AYCs’ situation, and subsequently enabling them to access tailored information, education, and support, as appropriate [7].

In addition, our study points to the need for staff development/educational initiatives at national, regional, and local levels for front-line practitioners and decision makers working directly with older people. In particular, with a focus on family caregiving across the life course [72], and with specific attention given to staff awareness raising of the particular situation of (A)YCs of older people.

## 5. Conclusions

The phenomenon of AYCs is still hidden, but quite widespread in Europe, and the topic of AYCs of GrPs is particularly under-researched by scholars. Our cross-national study highlights that AYCs of GrPs experienced more positive feelings in connection to their caring role, in comparison to AYCs of ORCs. This finding is seen to be related to the quality of the relationship between carer and care recipient, more than on the kinship relationship itself, given the lack of statistically significant differences between AYCs of GrPs, and of OCRs, in the MACA-YC18. Nevertheless, an excessive number of caring hours can cancel the mitigating effect given by the close relationship of adolescents with GrPs. AYCs (of GrPs) living in families who could count on formal care services were more exposed to the risk of health problems, and to negative caregiving outcomes, likewise there was also a significant effect of formal services on negative outcomes of caring. These are probably due to the severe health conditions of GrPs entitled to receive formal support, especially in countries without fully developed LTC systems.

More research involving large samples of AYCs is needed, for identifying drivers of caregiving for GrPs, and its consequences on specific aspects of health, e.g., mental well-being, social inclusion, education, and employment, in order to design policies, services, and practices that can minimize the negative impacts and valorize the positive impacts (e.g., self-esteem) of this form of intergenerational solidarity. The main goal for health and social care practitioners and policy makers, indeed, should be to help AYCs preserve the intergenerational emotional bonds with older family members, whilst protecting them from negative outcomes, resulting from inappropriate responsibilities that might undermine their general health and well-being.

## Figures and Tables

**Table 1:** Sample characteristics according to the care recipients: AYCs of other care recipients (OCRs) and AYCs of grandparents (GrPs).

	AYCs			*p*
	Total	OCRs	GrPs	
	817 (100%)	679 (83.1%)	138 (16.9%)	
Gender				0.536
Male	202 (24.7%)	173 (25.5%)	29 (21.0%)	
Female	591 (72.4%)	486 (71.5%)	105 (76.1%)	
Transgender/non-binary	24 (2.9%)	20 (3.0%)	4 (2.9%)	
Age				0.104
15	104 (12.7%)	94 (13.8%)	10 (7.2%)	
16	361 (44.2%)	297 (43.8%)	64 (46.4%)	
17	352 (43.1%)	288 (42.4%)	64 (46.4%)	
Country of birth %				0.631
National	752 (92.5%)	623 (92.3%)	129 (93.5%)	
Abroad	61 (7.5%)	52 (7.7%)	9 (6.5%)	
Formal support services received by AYCs’ family				0.005
No	394 (49.1%)	310 (46.6%)	84 (61.8%)	
Yes	296 (36.9%)	260 (39.0%)	36 (26.5%)	
I do not know	112 (14.0%)	96 (14.4%)	16 (11.8%)	
Hours a day spent providing care	3.9 ± 5.0	4.0 ± 5.2	2.9 ± 3.6	0.014
Country of residence				
CH	40 (4.9%)	31 (4.6%)	9 (6.5%)	<0.001
IT	116 (14.2%)	66 (9.7%)	50 (36.3%)	
NL	79 (9.7%)	66 (9.7%)	13 (9.4%)	
SE	280 (34.3%)	266 (39.2%)	14 (10.1%)	
SL	135 (16.5%)	96 (14.1%)	39 (28.3%)	
UK	167 (20.4%)	154 (22.7%)	13 (9.4%)	

Note: data are n (%) where not specified.

**Table 2:** Outcome variables per care recipients: AYC of OCRs and AYCs of GrPs.

	Range	AYCs			*p*
		Total	OCRs	GrPs	
		817 (100%)	679 (83.1 %)	138 (16.9%)	
MACA-YC18	1–36	13.2 ± 5.7	13.3 ± 5.7	12.8 ± 5.6	0.326
PANOC-YC20 Negative Outcomes	0–20	5.6 ± 5.2	5.8 ± 5.2	4.2 ± 4.9	0.001
PANOC-YC20 Positive Outcomes	0–20	12.7 ± 47	12.5 ± 4.6	13.8 ± 4.8	0.003
KIDSCREEN-10 General health status	10–50	32.9 ± 7.3	32.9 ± 73	33.2 ± 7.2	0.603
Health problems in connection with caring role, n (%)					<0.001
No		468 (57.3%)	384 (56.6%)	84 (60.9%)	
Yes		349 (42.7%)	295 (43.4%)	54 (39.1%)	

Note: data are mean ± sd where not specified. Statistical significance was tested by t test or chi squared as appropriate.

**Table 3:** Exposure factors to positive and negative caregiving outcomes and health problems among AYCs of OCRs and AYCs of GrPs (n = 817).

	PANOC-YC20 Negative Outcomes		PANOC-YC20 Positive Outcomes		Health Problems in Connection to Care Role	
	Β	*p*	β	*p*	OR	*p*
Caring for GrPs (ref. No)	−1.09	0.010	1.20	0.008	0.94	0.765
Age (ref.15)						
16	−0.76	0.159	0.49	0.330	0.76	0.245
17	−0.75	0.157	0.68	0.179	0.77	0.278
Gender (ref. Male)						
Female	0.90	0.021	0.41	0.275	1.53	0.016
Transgender/non-binary	3.50	0.006	−1.76	0.127	4.71	0.002
Hours a day spent providing care	0.22	<0.001	0.01	0.721	1.07	<0.001
Country of birth (ref. National)						
Abroad	2.49	<0.001	0.36	0.560	1.30	0.382
Formal services (ref. No)						
Yes	1.86	<0.001	−0.38	0.292	1.79	<0.001
Don’t know	1.38	0.007	−0.58	0.256	0.84	0.457
Constant	3.71	<0.001	11.89	<0.001	0.43	0.003
R^2^	0.139		0.025		0.059	

Notes: data are coefficients (β), odds ratios (OR) and level of statistical significance (*p*). Robust standard errors were applied. Mean variance inflation factors (VIFs) for the models was 1.34 (range: 1.02–2.58).

**Table 4:** Exposure factors to positive and negative caregiving outcomes and health problems among AYCs of OCRs and AYCs of GrPs (n = 817) when country is included in the regression model.

	PANOC-YC20 Negative Outcomes		PANOC-YC20 Positive Outcomes		Health Problems in Connection with Caring Role	
	Β	*p*	β	*p*	OR	*p*
Caring for GrPs (ref. No)	0.21	0.619	0.24	0.604	0.95	0.786
Age (ref.15)						
16	−0.33	0.520	−0.15	0.769	0.71	0.158
17	−0.41	0.422	0.10	0.837	0.71	0.160
Gender (ref. Male)						
Female	1.19	0.002	0.14	0.708	1.51	0.022
Transgender/non-binary	3.58	0.003	−1.68	0.128	5.02	0.002
Hours a day spent providing care	0.20	<0.000	0.01	0.829	1.06	0.002
Country of birth (ref. National)						
Abroad	2.13	0.001	0.53	0.382	1.28	0.407
Formal services (ref. No)						
Yes	1.16	0.002	0.25	0.487	1.71	0.002
Don’t know	0.39	0.472	0.01	0.989	0.94	0.801
Country (ref. CH)						
IT	−3.75	<0.001	1.64	0.069	0.56	0.153
NL	−3.40	0.001	−1.23	0.224	0.37	0.024
SE	−0.14	0.895	−0.89	0.303	0.52	0.092
SL	−3.87	<0.001	2.04	0.022	0.59	0.184
UK	−0.05	0.963	−0.88	0.331	0.83	0.646
Constant	4.99	<0.001	12.50	<0.001	0.82	0.654
R^2^	0.237		0.085		0.068	

Notes: data are coefficients (β), odds ratios (OR) and level of statistical significance (*p*). Robust standard errors were applied. Mean variance inflation factors (VIFs) for the models was 2.25 (range: 1.03–5.76).
